# The Potential of Ozone Therapy as an Adjunct to Periodontal Treatment in Diabetes Mellitus: A Biomarkers Scoping Review

**DOI:** 10.2174/0115733998353396250416114716

**Published:** 2025-05-08

**Authors:** Elisa Grillo Araújo, Domitilla Marchiori Sant’Anna Leal de Oliveira, Sérgio Bruzadelli Macedo, Cristine Miron Stefani, Loise Pedrosa Salles

**Affiliations:** 1 Post-Graduation in Dentistry, Faculty of Health Sciences, University of Brasilia, Brasilia, Brazil;; 2 Department of Dentistry, Faculty of Health Sciences, University of Brasilia, Brasilia, Brazil

**Keywords:** Ozone, diabetes mellitus, periodontitis, periodontal therapy, biomarkers, immunomodulation

## Abstract

**Introduction:**

Periodontitis is a prevalent inflammatory disease and a critical complication in individuals with Diabetes Mellitus (DM). The bidirectional relationship between periodontitis and DM exacerbates systemic inflammation and complicates disease management. Ozone therapy (OT), with its antimicrobial and immunomodulatory properties, has emerged as a promising adjunctive treatment for periodontitis in individuals with DM, though it remains underexplored. This scoping review evaluates the effects of OT on biomarkers associated with DM, which may indirectly suggest its potential as a complementary therapy for periodontitis management in DM.

**Methods:**

A comprehensive search of PubMed, Cochrane, Embase, LIVIVO, Scopus, Web of Science, and LILACS (up to March 2024) identified 38 studies: 19 involving individuals with DM and 19 using DM animal models.

**Results and Discussion:**

OT positively influenced biomarkers such as Malondialdehyde (MDA), Superoxide Dismutase (SOD), reduced Glutathione (GSH), Catalase (CAT), Total Antioxidant Status (TAS), and Total Oxidant Status (TOS). OT also modulated inflammatory markers (TNF-α, IL-1β, IL-6, IL-8, IL-10, and CRP) and regenerative biomarkers (bFGF, BMP-2, CD34, VEGF, PDGF) while improving glycated hemoglobin (HbA1c). This study represents the first review to explore the effects of OT on DM biomarkers and infer possible implications of these effects if used as an adjunct in the treatment of periodontitis associated with DM.

**Conclusion:**

OT may complement conventional periodontal treatments in individuals with DM by modulating biomarkers linked to periodontal health. These insights may guide future clinical studies to validate OT’s role in the treatment and establish standardized protocols for its application in periodontal management in DM.

## 1 INTRODUCTION

Ozone therapy (OT) is a complementary medical treatment that uses a mixture of oxygen and ozone as a therapeutic agent for various diseases [1], demonstrating notable results in accelerating the healing process of chronic wounds [2-4], combating infections [5-7], and managing inflammatory diseases [8-10]. Due to its antimicrobial and immunomodulatory effects, OT has the potential to serve as an adjuvant for treating periodontitis in Diabetes Mellitus (DM). However, despite its promise, research in this specific area remains limited, highlighting a significant gap in the literature.

Ozone, a highly reactive form of oxygen, exhibits antimicrobial activity and modulates the immune response by generating a diverse array of bioactive compounds that trigger various molecular mechanisms [11-13]. OT, which involves the calibrated and transient application of ozone, induces oxidative eustress, potentially promoting tolerance to Reactive Oxygen Species (ROS). The concept of oxidative eustress refers to a controlled level of oxidative stress that activates beneficial cellular signaling pathways without causing cellular damage [14, 15]. This mechanism of action allows the organism to develop an adaptive response that increases tolerance to higher levels of ROS and enhances its ability to manage oxidative stress [16-19]. Additionally, OT reduces inflammation, improves circulation, and promotes the release of autacoids and growth factors [20-22] (Fig. **1**).

DM encompasses a group of metabolic disorders with an exponential global prevalence [23, 24]. It is characterized by hyperglycemia resulting from defects in insulin secretion, insulin action, or both [25]. The oxidative stress and inflammation triggered by hyperglycemia are pivotal factors in the progression of DM and contribute significantly to cellular damage in affected tissues [26-32]. Beyond periodontitis, DM manifests in a range of clinical conditions, including retinopathy, neuropathy, nephropathy, cardiovascular complications, and peripheral vascular diseases [23, 33]. These complications highlight the systemic burden of DM and the critical need for innovative therapeutic approaches [34, 35].

Although many studies have investigated biomarkers responsive to OT as an adjunct in the treatment of various DM complications, its potential as an adjunctive therapy, specifically for periodontitis in individuals with DM, remains insufficiently explored. This gap in knowledge underscores the necessity of examining indirect evidence from other tissues to better infer the effects of OT as an adjunctive treatment for periodontitis in individuals with DM.

This scoping review addresses the question: Does OT affect biomarkers that may indicate a potential benefit when used as an adjunct to treat periodontitis associated with DM? By exploring the potential impact of OT on periodontal treatment in individuals with DM, this review seeks to guide future research toward the development of therapeutic protocols aimed at achieving improved periodontal outcomes for these individuals.

## 2 MATERIALS AND METHODS

### 2.1 Protocol and Registration

This scoping review followed the Preferred Reporting Items for Systematic Reviews and Meta-Analyses extension for Scoping Reviews [36], and was registered in the Open Science Framework website (OSF, https://osf.io/) under the identifier doi: 10.17605/OSF.IO/FRGHV.

### 2.2 Search Strategy and Eligibility Criteria

PubMed (MEDLINE), Cochrane (CENTRAL), Embase, LIVIVO, Scopus, Web of Science, and LILACS databases were searched up to June 26, 2023, with an update conducted on March 19, 2024. The search utilized the main descriptors “ozone” and “diabetes mellitus” with no language or publication period restrictions. Additionally, we searched the grey literature using ProQuest (Dissertation and Theses) and Google Scholar. The complete search strategy for each database is available in Supplementary File **1**.

Inclusion criteria followed the PCC acronym: Population (P): people with DM (type 1 and type 2) and animal models for DM; concept (C): evaluating effects on biomarkers of OT (gas, water, or oil); context (C): in clinical and preclinical studies.

Exclusion criteria comprised: (1) studies of non-DM individuals or animals, (2) studies with no OT, (3) studies not evaluating biomarkers/effects as outcomes, (4) *in-vitro* studies, non-intervention studies, opinion articles, letters, and studies with no full text available.

Search results were organized in Mendeley Desktop (v.1.19.8, Elsevier, Amsterdam, The Netherlands) to remove duplicates. Then, two independent reviewers (EGA and DMSLO) assessed titles and abstracts using the Rayyan QCRI application [37] based on inclusion criteria, analyzed full texts to confirm eligibility, and resolved disagreements through consensus.

### 2.3 Data Extraction and Synthesis

Data extraction was performed independently by two reviewers (EGA and DMSLO). It included authorship, publication year, country, study design, sample characteristics, OT intervention details, biomarkers assessed, follow-up, and main findings. A descriptive narrative synthesis was conducted to summarize the results.

## 3 RESULTS

### 3.1 Characteristics of the Studies

The search identified 1.391 studies, of which 885 remained after removing duplicates. After analyzing titles and abstracts, 844 references were excluded. Forty-one (41) full texts were evaluated, resulting in the inclusion of 38 studies (Fig. **2**). The list of excluded articles, along with the reasons for exclusion, is available in Supplementary File **2**.

Of the 38 included studies, 19 were conducted in human subjects (Table **1**) between 2005 and 2024 in Cuba [38-40], Mexico [41], Iran [42, 43], Ukraine [44, 45], Italy [46], Albania [47, 48], Russia [49-51], China [52-54] and Turkey [55]. Notably, one study [56] did not specify the country of publication. These studies investigated the impact of OT on various health conditions related to DM. (Fig. **3**), including metabolic control in 122 DM individuals [38, 55]; oxidative damage progression in 15 DM individuals (type not specified) who also had rheumatoid arthritis and hypertension [39]; peripheral neuropathy in 25 T2DM individuals [41]; foot complications in 100 DM individuals (type not specified) [42], and 232 T2DM individuals [40, 50, 52-54]; soft tissue infections in 106 DM individuals (type not specified) [44, 45] and 48 T2D individuals [49, 51]; chronic periodontitis in 20 DM individuals (type not specified) [46] and 140 T2DM individuals [47, 48]; visual evoked potential in 60 T2DM individuals [43] and growth/ differentiation Factor-15 (GDF-15) in 21 T2DM individuals [55]. Finally, the effects of OT on nephropathy were investigated in 6 T2DM individuals [56].

The remaining 19 studies utilized rat models of DM (Table **2**). These investigations were conducted between 2001 and 2023 in Cuba [57, 58], Turkey [59-66], Egypt [67-70], the United States of America [71], China [72, 73], Indonesia [74] and Iraq [75]. The studies delved into the impact of OT on hyperglycemia [67, 69], endothelial damage [57], erectile dysfunction [60], neuropathy [61, 64, 66], oxidative stress in lung tissue [62], pancreas damage [58, 75], nephropathy [63, 68], retinopathy [72, 73], ulcer associated with DM [74], as well as the promotion of wound healing [65, 70, 71] and osseous healing [59].

### 3.2 Synthesis of Results

Although, only three studies specifically investigated the effect of OT on DM-associated periodontitis [46-48], all studies evaluated the effect of OT on biomarkers whose effects may infer benefits to periodontal tissues. Many of these biomarkers have already been associated with the periodontal context in DM (Table **3**).

#### 3.2.1 Antioxidant Effects

OT demonstrated significant effects on redox balance markers (Fig. **4**), notably reducing injury markers such as Malondialdehyde (MDA) [39, 57, 58, 68, 69, 75], Total Hydroperoxide (TH) [39] Plasma Oxidant Levels (TOS) [62, 64], Oxidative Stress Index (OSI) [62, 64], fructolysine [57], Advanced Oxidation Protein Products (AOPP) [39, 40, 57], Peroxidation Potential (PP) [40] and Nrf2 (nuclear-related factor 2)/ARE (Antioxidant Response Element) binding activity [34]. Additionally, it significantly augmented antioxidant defense markers, including Glutathione (GSH) [39, 40, 57, 58, 75], Superoxide Dismutase (SOD) [39, 40, 54, 57, 68, 75], Catalase (CAT) [39, 40, 57, 68, 75], Glutathione Peroxidase (GPx) [58, 68], Plasma Antioxidant Levels (TAS) 61, 62, 64], Endothelial Nitric Oxide Synthase (eNOS) [60] Salivary Antioxidant Test (SAT) [46], total Antioxidant Capacity (TAC) [69]. Furthermore, it effectively prevented the depletion of nitrites/nitrates (NO−2/NO−3) [57].

#### 3.2.2 Anti-glycemic Effects

OT resulted in a statistically significant decrease in glycemic markers (Fig. **4**), such as glycated hemoglobin (HbA1c) or fasting blood sugar (FBS), when compared to conventional treatments [38, 40, 41, 43, 45, 57, 58, 61, 66, 68-70].

#### 3.2.3 Anti-inflammatory Effects

OT exhibited reduction in inflammatory markers (Fig. **4**), including Tumor Necrosis Factor -α (TNF-α) [54, 63, 73], Interleukin-1 β (IL1- β) [73], Interleukin- 6 (IL-6) [51, 54, 73], Interleukin- 8 (IL-8) [49, 51], Interleukin- 10 (IL-10) [49, 51], C-Reactive Protein (CRP) [42, 54], and Erythrocyte Sedimentation Rate (ESR) [54]. Moreover, it normalized the total leukocyte count in wounds by decreasing neutrophils and increasing lymphocytes, monocytes, and macrophages [50].

#### 3.2.4 Healing Effects

OT also resulted in an impact on markers associated with the healing process (Fig. **4**). It led to an increase in heat shock protein 90α (HSP90α) [74], EGF [71, 74], basic Fibroblast Growth Factor (bFGF) [49, 51, 74], CD34 levels [74], Vascular Endothelial Growth Factor (VEGF) [52-54, 65, 70, 72-74], cell migration [71], Insulin-like Growth Factor 1 receptor (IGF1R) [71], Transforming Growth Factor (TGF) [52, 53, 65, 70], Bone Morphogenic Protein (BMP)-2 [59], Growth/Differentiation Factor-15 (GDF-15) [55], osteocalcin [59], Platelet-Derived Growth Factor (PDGF) [52, 53], potassium [56], creatine [56]. Additionally, OT exhibited a reduction in Hypoxia-Inducible Factor-1α (HIF-1α) [63, 72].

## 4 DISCUSSION

This scoping review provides a comprehensive compendium of the available evidence on the effects of complementary OT on biomarkers in treating DM and its clinical manifestations. The studies emphasize the impact of OT on cytokines, proteins, and cells associated with antioxidant, anti-inflammatory, and reparative effects, suggesting potential clinical benefits for OT when used as an adjuvant to periodontal treatment in individuals with DM (Fig. **4**).

While ozone’s well-established antimicrobial effects [18, 76-79] are pivotal in periodontal care, other less-explored effects, such as its potential to activate biological mechanisms enhancing tissue healing processes, may be equally or even more valuable than its antimicrobial properties, particularly in DM individuals.

The application of OT as an adjunct to periodontal treatment in individuals with DM has been evaluated in three studies [46-48]. These studies assessed plasma markers of oxidative stress and HbA1c levels, suggesting minimal systemic impacts of local ozone applications on periodontal tissues. However, further investigation is needed to better understand the local effects of OT on biomarkers related to periodontal status.

The local effects of OT on soft tissues have been studied in DM foot complications and wound healing. These studies demonstrate a significant reduction in wound size and faster healing. The findings are supported by a systematic review on chronically refractory wounds and ulcers [80]. The increase in growth factors observed in these studies is likely a key contributor to these results. Growth factors play a crucial role in periodontitis associated to DM, given the metabolic and immune alterations associated with the disease. High levels of TNF in DM suppress proliferation of bone-lining cells due to the impact of reduced growth factors expression [81], aggravating periodontitis.

As evidenced by several included studies, OT has shown to promote the expression of various growth factors [49, 52-55, 65, 71, 72, 74], including bFGF, TGF, VEGF, IGF1R, BMP-2, PDGF and GDF-15. Particularly, when used as an adjunct to non-surgical periodontal in non-DM individuals, OT was associated with higher levels of TGF-β [97, 98]. These growth factors play a crucial role in accelerating healing, a vital process in combating periodontal complications in DM individuals. The elevation of VEGF, coupled with the simultaneous negative regulation of endothelin receptor A (ETAR), underscores the potential of OT to foster a favorable environment for tissue healing in DM [99].

The mechanism underlying the efficacy of OT in wound healing may possibly be explained by its potential activation of the PI3K/Akt/mTOR signaling pathway [100]. This pathway, renowned for its role in cellular functions such as proliferation, survival, and motility [101], warrants exploration as a mediator of the ozone therapeutic cascade. The activation of the PI3K/Akt signaling not only promotes epithelial migration and proliferation but also orchestrates macrophage polarization [102, 103], thus modulating the crucial immune response for periodontal health. Through the PI3K/Akt/mTOR pathway, OT may facilitate a transition from pro-inflammatory M1 macrophages to anti-inflammatory M2 phenotypes, providing a balanced immune and inflammatory response essential for periodontitis management.

Several adjuvant treatments for periodontitis under investigation have been proposed based on the action of substances capable of alleviating inflammatory cell infiltration by regulating macrophage polarization, which is activated by the PI3K/AKT/mTOR signaling pathways [104-106]. This suggests that the potential action of ozone on this pathway may lead to improved outcomes of periodontal therapy in DM individuals.

This review is the first to explore the effects of adjunctive OT on biomarkers in the treatment of DM manifestations, aiming to infer its potential benefits as an adjuvant to periodontal treatment. However, it has several limitations, primarily the scarcity of studies on OT in DM- associated periodontitis. As a scoping review, it did not include the assessment of effect sizes and risk of bias evaluations, which are crucial for a more detailed analysis of study quality and impact. Future studies should investigate OT specifically in DM-associated periodontitis. These studies should also focus on local biomarkers within periodontal tissues, involve larger sample sizes, and assess the economic impact on public health.

## CONCLUSION

The observed effects of OT on biomarkers, such as antioxidant enzymes, growth factors, and cytokines, suggest its promising potential as an adjunct to conventional treatment of periodontitis in individuals with DM, with special emphasis on the effects of local OT application on tissue repair biomarkers. Future studies are crucial to validate these findings within the periodontal context, further investigate the mechanisms of action in periodontal tissues, and establish standardized protocols to guide its clinical application.

## Figures and Tables

**Fig. (1):**
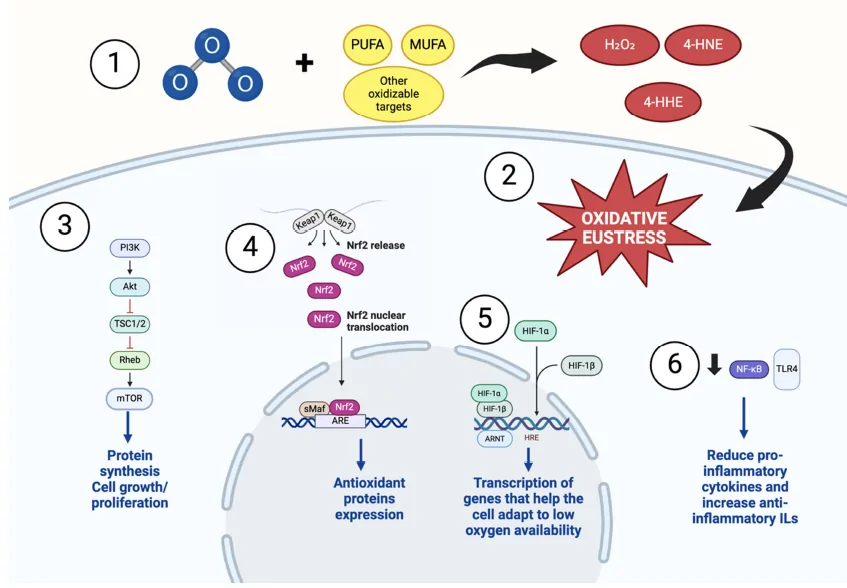
Mechanisms of OT action - (**1**) Reaction with Oxidizable Targets: Ozone reacts with oxidizable targets, such as polyunsaturated fatty acids (PUFAs) and monounsaturated fatty acids (MUFAs), generating reactive oxygen species (ROS), including hydrogen peroxide (H_2_O_2_), and lipid oxidation products (LOPs), such as 4-hydroxy-2-nonenal (4-HNE) and 4-hydroxy-2-hexenal (4-HHE) [12, 13]. (**2**) Induction of Oxidative Eustress: These reactive species induce a state of oxidative eustress, which activates protective and adaptive cellular pathways [13-15]. (**3**) Activation of the PI3K/AKT/mTOR Pathway: The phosphoinositide 3-kinase/protein kinase B/mechanistic target of rapamycin (PI3K/AKT/mTOR) pathway is activated, leading to increased protein synthesis, cell growth, and proliferation [102]. (**4**) Nrf2 Pathway Activation: Oxidative eustress triggers the release of nuclear factor erythroid 2-related factor 2 (Nrf2), which translocates to the nucleus and promotes the expression of antioxidant genes [12-14]. (**5**) Hypoxia Response through HIF-1α Activation: Activation of hypoxia-inducible factor 1-alpha (HIF-1α) supports the transcription of adaptive genes that enhance oxygen availability under hypoxic conditions [12, 14]. (**6**) Regulation of NF-κB Activity: Reduced activity of nuclear factor kappa B (NF-κB) decreases pro-inflammatory cytokines and increases anti-inflammatory interleukins (ILs), contributing to cellular homeostasis [14, 15].

**Fig. (2):**
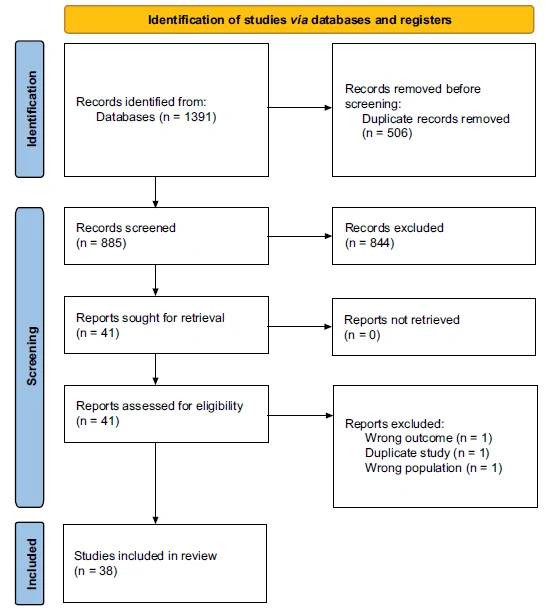
Flow diagram of the inclusion process according to the PRISMA statement.

**Fig. (3):**
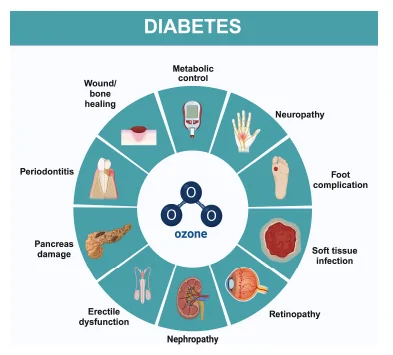
Complications of diabetes treated with adjuvant ozone therapy related to studies in this review.

**Fig. (4):**
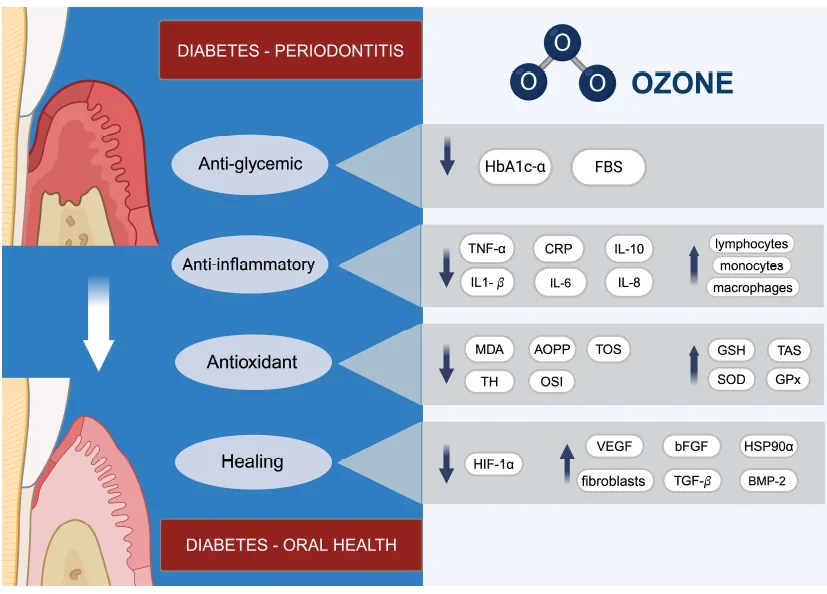
Potential clinical benefits of ozone when used as an adjuvant to periodontal treatment in individuals with DM, involving anti- glycemic, anti-inflammatory, antioxidant, and healing effects.

**Table 1:** Summary of descriptive characteristics of included human articles (n=19).

**Authorship** **Year** **Country**	**Study design**	**Investigated condition**	**Participants (n)**	**Interventions/OT protocol**	**Biomarker assessed**	**Follow Up**	**Main Findings**
Barroso *et al.*, 2023 [38] Cuba	Quasi-experimental study	DM	101 DM individuals (95% T2D)	Conventional therapy (hypoglycemic) + Rectal insufflations-20 daily sessions in escalating doses: 20 μg/ml in 100 ml, 25 μg/ml in 120 ml, 30 μg/ml in 150 ml and 35 μg/ml in 200 ml	Blood glucose levels	12 months	2,769 mmol/L decrease in blood glucose levels: (Before intervention: 11,104 mmol/L, after: 8.335 mmol/L).
Fernández *et al.*, 2022 [39] Cuba	RCT	Oxidative damage progression in elderly individuals with oxidative etiology diseases	45 older individuals (60-70 years) Group I (n=15) had no ozone and Group II (n=30) ozone, which was divided into 2 subgroups: Group IIa (n=15) DM individuals with rheumatoid arthritis and hypertension; Group IIb (n=15) with asthma	Treatment of associated diseases + Rectal insufflations-20 treatments. Next, 20-30 mg/l of ozone, administered in a stepped application and an increasing order: First week: 20 mg/l, 100 ml; second: 25 mg/l, 100 ml; third: 25 mg/l, 150 ml; and fourth: 30 mg/l, 200 ml	AOPP (Advanced oxidation protein products), GSH, SOD, CAT (Catalase activity), MDA, TH (Total hydroperoxides), TXA_2 (_Thromboxane A2), and PGI2 (Prostacyclin)	3 weeks	AOPP- significantly decreased. Comparing redox biomarkers between group I and group II, statistical differences were found. Antioxidant defenses (GSH, SOD and CAT) increased, while injury biomarkers (MDA and TH) decreased.
Guadalupe *et al.*, 2023 [41] Mexico	Clinical trial	Peripheral neuropathy (DNP)	25 T2D with peripheral neuropathy	Initial management (glycemic control, administration of analgesics, non-steroidal anti-inflammatory drugs and tricyclic Antidepressants) + Rectal insufflations (27 ug/ml) in 12 sessions for 35 days-applied every third day	HbA1c, glucose, urea, creatinine, urea nitrogen, albumin, cholesterol, lipid profile, oxidative damage in lipids, antioxidant capacity and Nrf2/ARE (Nuclear-related factor 2)	6 months	Antioxidant capacity decreased (no biological plausibility), plasma oxidative damage reduced by 68% and the Nrf2/ARE binding activity showed a 10% decrease *i.e.*, 0.20±0.04(*p*=0.013).
Izadi *et al.*, 2019 [42] Iran	Single-blind RCT	Foot ulcer	200 T1D and T2D, divided into two groups: group 1: ozone and conservative treatment, group 2 just routine cares	Routine care + Local ozone (gas and oil) and systemic (minor and major OT)-twice a week. Local ozone: bag (gas) for 30 min, ozonated gel (Ozolive) on the wounds every 12 h, subcutaneous injection around the wound. Systemic ozone: rectal or intravenous	FBS (Fasting blood sugar), CRP, and ESR (Erythrocyte sedimentation rate)	19 months	In ozone group not only all variables were decreased but also it's after treatment values were less than those in control group.
Izadi *et al.*, 2023 [43] Iran	Single-centre, randomized, controlled, parallel-group study	Visual evoked potentials	60 T2D, divided into two groups-group 1: ozone and standard DM therapy, group 2 just standard DM therapy	Standard DM therapy + Major and minor autohemo-therapy was performed twice a week for 20 sessions, 10 weeks in total	HbA1c and Visual evoked potentials (VEP) - P100 latency and amplitude	3 months	Ozone therapy reduced P100 wave latency and improved glycemic control in DM individuals.
Karatieieva *et al.*, 2017 [44] Ukraine	Clinical trial	Soft tissue complications	124 DM (type not specified): 53-OT, 71-conventional methods	Conservative and surgery treatment (antibiotics, infusion therapy, antiplatelet agents, antispasmodics, vitamins, insulin, and others) + Intravenous ozone saline for 6-15 days	LPO (Products of lipid peroxidation), OMP (Oxidative modification of proteins), AOP (Antioxidant protection), LII (Leucocyte index of intoxication), HTI (Hematological toxicity index), and SCE (Sorption capacity of red blood cells)	7 and 16 days	No significant differences in the activity of AOP and OMP. Significant difference regarding LII, HTI and SCE.
Karatieieva *et al.*, 2018 [45] Ukraine	Clinical trial	Soft tissue complications	124 DM individuals (type not specified), 2 groups: severe stage (n= 76) and moderate stage (n= 48). Enrolled individuals were divided into 2 groups: ozone (n=53) and control (n= 71): only conventional treatment	Conventional treatment + Intravenous ozone saline, 6 day-period. Individual doses of ozone were determined using a lymphocytotoxic test	Glucose level and leukocyte index of intoxication	3, 6 and 14 days	After 6 days, glucose decreased, and clinically significant difference was observed between the groups. After 14 days, glucose levels decreased in both groups - similar values.
Marconcini *et al.*, 2021 [46] Italy	RCT	Chronic periodontitis	20 type 1 or type 2 DM	Antioxidant mouthwash or chlorhexidine + domestic use of an ozone delivery device (ozoned water) twice a day	dROMs (Reactive oxygen metabolites), PAT (Plasmatic antioxidant test), SAT (Salivary antioxidant test) and HbA1c	1, 3 and 6 months	Reduction in SAT at the 3-month. No statistically significant results were found after 6 months follow-up.
Martínez-Sánchez *et al.*, 2005 [40] Cuba	RCT	Neuroinfectious foot	100 T2D 49-topical and systemic antibiotic (conventional method of treatment) and 51-ozone	Debridement + Rectal insufflations and local ozone-20 daily sessions 10 mg, 50 mg/l. Local ozone-lesion covered with a plastic bag. Afterward, the bag was refilled with ozone, 60 mg/l for 1 h. After that, the bag was removed, and the lesion was covered with ozonized sunflower oil (Oleozon®)	Glucose levels, serum levels of fructolysine, AOPP (advanced oxidation protein products), nitric oxide, GSH, GPx (glutathione peroxidase), CAT (catalase), SOD, total hydroperoxides, PP (peroxidation potential) and MDA	20 days after treatment	Glucose, AOPP, PP and CAT/SOD ratio significantly decreased in the ozone group. GSH: lower in the antibiotic group but not changed in the ozone group. Enzyme activity significantly increased in the ozone group.
Qin *et al.*, 2020 [52] China	RCT	Foot ulcer	100 T2D individuals were randomized into 2 groups	50 individuals - local saline solution for cleaning + bag for ozone baths in the affected limb, 35 mg/L of ozone, for 30 minutes	Neutrophil percentage, CRP, ESR (Erythrocyte sedimentation rate), procalcitonin level, VEGF (Vascular endothelial growth factor), TGF-β and PDGF (Platelet-derived growth factor)	12 months	The expression levels of VEGF, TGF-β and PDGF in local wound tissues were significantly higher in the experimental group than in the control group (*p* < 0.05).
Rapone *et al.*, 2020 [47] Albania	RCT	Periodontitis	100 T2D-50 ozone and 50 conventional therapies	Periodontal therapy + Gaseous ozone after SRP, 2100 ppm with 80% oxygen four times, 30 s (every third day), 1 week	HbA1c	3, 6, 9 and 12 months	At 3 and 6 months, ozone group - slight reductions in HbA1c.
Rapone *et al.*, 2023 [48] Albania	Prospective, controlled, one-blind, RCT	Periodontitis	180 T2D: Group A (n=90): non-surgical periodontal treatment + OT. Group B (n=90): non-surgical periodontal treatment	Non-surgical periodontal treatment + Ozonated water (rinse/irrigation) and gas (2 cycles of ozone gas at 8-10 power)-4 sessions	Oxidative stress assessment: GSH, MDA, TAOS (Plasma levels of antioxidant levels), and TOS (Total Oxidant Status)	3 and 6 months	Ozone did not enhance the effect of periodontal treatment in reducing OS in plasma levels of periodontitis individuals with T2D.
Sherov, 2022 [56] (Country not reported)	Clinical assessment	Nephropathy	6 T2D with arterial hypertension and renal anemia	Angiotensin-converting enzyme inhibitors (ACE inhibitors) in combination with calcium blockers and recombinant erythropoietin preparations. + 25 mg/l gas ozone (missing protocol details)	Creatine, potassium and GFR (Glomerular filtration rate)	10 days after treatment	Normalized potassium and reduced creatinine.
Sun *et al.*, 2024 [54] China	Retrospective non-randomized study	DM foot ulcers	89 T2D divided into two groups: ozone therapy group (n=41) and control group (n=48)	Basic wound treatment + Ozone bag (35 mg/ml) for 30 minutes once a day	Wound condition, change of bacterial types, changes in inflammatory indicators (ESR, CRP, procalcitonin [PCT]), VEGF, IL-6 TNF- α, and oxidative stress levels (SOD, MDA, T-AOC)	1 week and 12 weeks	After 1 week of treatment, the ozone therapy group showed higher VEGF, SOD, and T-AOC levels compared to the control group (*p*<0.05), while CRP, PCT, ESR, IL-6, TNF-a, MDA levels and bacterial types were lower (*p*<0.05). And higher wound healing rate after 12 weeks.
Teplyakova *et al.*, 2022 [49] Russia	Prospective non-randomized study	Acute soft-tissue infections	58 T2D Basic treatment (BCT): n=30; OA group: BCT + ozonetherapy	BCT (systemic antibiotic, glucose management, and wound sanitation/dressings) + Ozonated autohaemotherapy (OA) - The dose of ozone delivered amounted to 1 mg. OA sessions were carried out from the first day of the postoperative period through days 7-9.	IL-6, IL-8, IL-10, TNF-α and bFGF	9 days	OA decreased, overproduction of IL- 8, 10 and induction of expression of bFGF.
Teuvov *et al.*, 2017 [50] Russia	Clinical trial	Foot syndrome	37 T2D-20 traditional treatments, 17 local and general ozone	Traditional surgical intervention + Intravenous and topical ozone: 200 ml of ozone-saturated 0.9% sodium chloride solution injected intravenously daily, 800-1000 mcg/L. Postoperative period-local ozone included daily dressings of wounds and the introduction of ozonized saline 3.3-4 mg/L in drains	Cellular composition of smears (Neutrophils, Macrophages, Fibroblasts)	1, 3, 5 and 7 days	On the 3^rd^-5^th^ day, the individuals of the main group showed normalization of leukocyte, decrease in the content of stab and segmented leucocytes, increase in lymphocytes and monocytes.
Vinnik *et al.*, 2015 [51] Russia	Clinical trial	Soft tissues infections	40 T2D: 20 received local ozone and 20 local and systemic ozone therapies	Traditional basic treatment (hypoglycemic and surgical treatment of wounds) + Local: 60 mg/l for 25 to 30 min in special closed environment plastic chambers. Autohemotherapy: 10 mg/L ozone	IL-6, IL-8, IL-10, TNF-ÿ and bFGF	24 months	Decrease of IL-10, IL-6. and IL-8, and consistently high level of bFGF production.
Yücel and İlanbey, 2023 [55] Turkey	Pilot study	GDF-15	21 T2D and 14 healthy volunteers	10 sessions of major autohemotherapy at a concentration of 25-35 micrograms/milliliter twice a week	GDF-15, fasting glucose, HbA1c, and lipid panel levels	Before and after major autohemotherapy sessions	GDF-15 and LDL-C levels were reduced after major autohemotherapy.
Zhang *et al.*, 2014 [53] China	RCT	Foot ulcer	50 T2D divided into 2 groups	Debridement once every two days and wound dressings + Ozone bag-25 individuals (ozone group) received 52 µg/mL ozone (total volume: 20-50 mL) in a special bag for 30 min per day for 20 days in addition to standard treatment	VEGF, TGF-β, and PDGF Levels	11 and 20 days	Expressions of VEGF, TGF-β, and PDGF proteins at day 11 were significantly higher in ozone group.

**Abbreviations:** bFGF: Fibroblast growth factor, CRP: C-reactive protein, DM: Diabetes Mellitus, ESR: Erythrocyte sedimentation rate, GDF-15: Growth/differentiation factor-15, GSH: Glutathione, HbA1c: Glycated hemoglobin, IL-6: Interleukin-6, IL-8: Interleukin-8, IL-10: Interleukin-10, LDL-C: Low- density lipoprotein cholesterol, MDA: Malondialdehyde, OS: oxidative stress, OT: Ozone therapy, PDGF: Platelet-derived growth factor, RCT: Randomized clinical trial, SOD: Superoxide dismutase, T2D: Type 2 diabetes mellitus, T1D: Type 1 diabetes mellitus, TAC: Total antioxidant capacity, TGF: Transforming growth factor, TNF- α: tumor necrosis factor-alpha, TNF-ÿ: Tumor necrosis factor ÿ, VEGF: Vascular endothelial growth factor.

**Table 2:** Summary of descriptive characteristics of included animal model articles (n= 19).

**Authorship, Year Country**	**Investigated Condition**	**Animal Model (n)**	**Interventions/OT Protocol**	**Biomarker Assessed**	**Follow Up**	**Main Findings**
Al-Dalain *et al.*, 2001 [57] Cuba	Endothelial damage	50 rats divided into 5 groups (n=10 each): (1) Control-treated only with physiological saline solution; (2) positive control-DM inducted; (3) ozone; (4) oxygen, one per day, as in group 3 but using oxygen only; (5) control ozone group, as group 3, but non-DM	Rectal insufflation-50 μl/ml. 10 treatments (1.1 mg/kg), one per day	SOD, GSH-Px, MDA, GSH, AOPP, aldose reductase, fructolysine, and NO_2_/NO_3_	11 days after STZ-induced DM and 24 h after the last treatment	Glycemic control improved, GSH, SOD and CAT levels increased, total number of peroxides and MDA reduced. Aldose reductase and fructolysine reduced while AOPP were not increased. NO_2_/NO_3_ were maintained without changes regarding non-DM control.
Al-Rumaidh and Hasan, 2023 [75] Iraq	Blood oxidative stress and pancreas damage	48 rats were randomly divided into six groups: Group I-Control, Group II Medical Ozone (MO), Group III (DM), GroupIV (DM+MO), GroupV (DM+Insulin), Group VI (DM+MO +Insulin)	Group Control: citrate buffer (0.1 M, pH 5) daily for six weeks. Group II (MO): 1.1 mg/kg of ozone, daily for six weeks. Group III (DM): citrate buffer (pH 5). Group IV (DM+MO): 1.1 mg/kg of ozone, daily for six weeks. GroupV (DM+Insulin): Insulin 0.75 IU/100 g daily for six weeks. Group VI (DM + MO + Insulin): 1.1 mg/kg of ozone and Insulin 0.75 IU/100 g daily for six weeks	Serum glucose concentration, histopathological examination of pancreas tissue, Enzymatic Antioxidants, Catalase (CAT), SOD, MDA	6 weeks	CAT, SOD and GSH significant increased and MDA decreased in the (MO) group compared to control group. (MO) group showed normal pancreatic parenchyma, (DM+MO) group displayed nearly normal structure with some focal necrosis of islets of Langerhans and (DM+MO+Insulin) group showed marked improvement with Langerhans normal pancreatic parenchyma; normal islets of Langerhans and normal acinar cell.
Alpan *et al.*, 2016 [59] Turkey	Osseous healing in DM	56 rats, divided into groups: 1) empty defect (control, n=14); 2) xenograft (XG, n=14); 3) empty defect treated with OT (control + ozone, n=14); and 4) xenograft and ozone application (XG + ozone, n=14)	Gaseous ozone (30 seconds before the periosteum was closed.) and daily (90 seconds over the scalp for 2 weeks (140 ppm at 2 L/d, 2.24 mg)	Osteocalcin and BMP-2 (bone morphogenic protein-2) expression	4 and 8 weeks	Osteoclast numbers in the XG + ozone group significantly decreased at week 8 compared with week 4, and higher than those of the other groups at week 4. The XG + ozone group showed stronger positive staining for BMP-2 cells, more osteoblasts in the mesenchymal tissue and strong positive expression for osteocalcin.
Colakerol *et al.*, 2020 [60] Turkey.	Erectile dysfunction (ED)	18 rats divided into three groups (n=6 per group): control; DM; DM + OT	Intraperitoneal injection-60 μl/ml of ozone, administered with a 25 G injector every day for 2 weeks	NO (Nitric Oxide), nNO (neuronal Nitric Oxide), eNO (endothelial Nitric Oxide) and iNO (inducible Nitric Oxide)	10 weeks	nNOS and iNOS: No statistically significant difference between the groups. However, eNOS was significantly higher in the rats of DM + OT group compared with others.
Erken *et al.*, 2015 [61] Turkey	Neuropathy (DN)	36 rats divided into 6 groups (n=6): control (C), ozone (O), DM (D), ozone-treated DM (DO), insulin-treated DM (DI), and ozone- and insulin-treated DM (DOI)	Intraperitoneal injection-50 μg/ml of ozone, given to the O, DO, and DOI groups (1.1 mg/kg)-1 min injection period once a day for 15 days. DOI group also received insulin.	TOS (Total Oxidant Status), eNOS(Total Antioxidant Status), OSI and blood glucose	4 weeks	Ozone reduced the blood glucose and HbA1c levels, and partially prevented DN, indicating that its neuroprotective effect in DN is mediated through OS, vascular and apoptotic mechanisms.
Erken *et al.*, 2016 [66] Turkey	Neuropathy (DN)	Rats with induced DM (number not informed). 6 groups (n=7): control (C), ozone (O), DM (D), ozone-treated DM (DO), insulin-treated DM (DI), and ozone and insulin-treated DM (DOI)	Intraperitoneal injection-50μg/ml of ozone was given to the O, DO, and DOI groups (1.1mg/kg)-1 min injection period once a day for 15 days. DOI group also received insulin	Blood glucose, HbA1c, plasma total oxidant status, OSI, and apoptotic cell number	6 weeks	Blood glucose, HbA1c, plasma total oxidant status, OSI and apoptotic cell number of the DO, DI and DOI groups were significantly lower than the D group.
Erken *et al.*, 2016 [62] Turkey	Oxidative stress (OS) in lung tissue	42 rats were divided into 6 groups (n=7): Control (C), ozone (O), DM (D), ozone-treated DM (DO), insulin-treated DM (DI), and ozone- and insulin-treated DM (DOI)	Intraperitoneal injection-1.1 mg/kg (50 μg/ml), once a day. Ozone was given to the O, DO, and DOI groups for 6 weeks. DOI group also received insulin	OS tests: OSI, TAS (total antioxidant status), and TOS (total oxidant status)	6 weeks	Increase in the TAS and a decrease in the TOS and OSI levels of DM rats. Although, the effect of combined ozone and insulin therapy on TAS, TOS, and OSI was higher than that of the ozone-only treatment, this difference was not significant.
Güçlü *et al.*, 2015 [63] Turkey	Nephropathy (DNP)	42 rats, randomly divided into six groups (n=7): control (C), ozone (O), DM (D), DM ozone-treated (DO), DM insulin-treated (DI), and ozone- and DM insulin-treated (DOI)	Intraperitoneal injection-50μg/ml of ozone was given to the O, DO, and DOI groups (1.1mg/kg, 1-min injection period once a day for 6 weeks). DOI group also received insulin	Caspase-1-3-9, HIF-1α, TNF-α (Tumor necrosis factor alpha) and glucose levels	6 weeks	Significantly decreased gene expressions of caspases-1-3-9, HIF-1α, and TNF-α and improved histopathological outcomes of the DNP. Apoptotic cell number downregulated in DOI
Helal *et al.*, 2013 [67] Egypt	DM	Twenty rats were divided into two groups: group 1 (control) and group 2 (induced DM rats). Group 2 was divided into three subgroups. SG1: DM untreated rats, SG2: DM treated with ozone and SG3: DM treated with vitamin C	Intraperitoneal injection of ozone, 50 μg/cm^3^, three times/week	Blood glucose, insulin, glycogen content (mg/dl), HOMA-IR, lipid profile (VLDL, HDL and LDL), total protein level, albumin, globulin, ALAT (alanine aminotransferase), ASAT (aspartate aminotransferase), creatine, urea, hematological parameters, and histological changes	1 month	O3 and vitamin C improved serum insulin level, significantly decreased blood glucose and did not change HOMA-IR in comparison to the control group.
Hesham *et al.*, 2023 [70] Egypt	Wound healing	64 rats were randomly divided into two groups: ozonated water group (O3W) and control group (CG)	Irrigation with normal saline (CG) or intradermally injected with 1 mL of ozonated water at 10 mg/L O3W	Wound healing, VEGF and TGF	3, 7, 9, 12, 14, 18, 21, 24, and 28 days post-wounding	O3W group had significantly lower blood glucose levels and woundsize, and had gained body weight. Additionally, epithelial vascularization, stromal edema, TGF, and VEGF gene expression significantly improved in the O3W group.
Karademir *et al.*, 2018 [64] Turkey	Cranial and spinal neuropathy	60 rats divided into six groups (n=10): control (C), ozone (O), DM (D), DM ozone-treated (DO), DM insulin-treated (DI), and DM ozone + insulin-treated (DOI)	Gas-50 μg/ ml of ozone, 1.1 mg/kg ip; 1-min injection once a day. DOI group also received insulin	TOS (total oxidant status), TAS (total antioxidant status), and OSI	60 days	TOS and OSI significantly decreased in DO, DI, and DOI groups, compared with the D group. Differences observed regarding TAS and TOS levels among DO, DI and DOI were not significant.
Martinez *et al.*, 2005 [58] Cuba	Oxidative stress and pancreas damage	50 rats, divided into 5 groups (n=10 each): Group 1: no DM, physiological saline solution; Group 2: DM induced; Group 3: DM, Ozone; Group 4: DM, oxygen; Group 5: No DM, ozone	Rectal insufflation-50μl/ml. 10 treatments (1.1 mg/kg), one per day	SOD, CAT (catalase), GSH-Px, MDA, GSH and Total protein concentration	11 days after DM induction	Ozone significantly increased GPx and reduced MDA. O3 also reduced hyperglycemia and improved lipid peroxidation products.
Morsy *et al.*, 2010 [68] Egypt	Nephropathy (DNP)	60 rats, divided into 6 groups: Control (C); Ozone (O); Untreated DM (D); DM Rats + insulin (DI); DM rats + ozone (DO); Group DM rats + insulin + ozone (DIO)	Intraperitoneal injection-50 μg/ ml of ozone. The DIO group also received insulin.	HbA1c, serum creatinine, blood urea nitrogen (BUN), kidney tissue levels of SOD, CAT (Catalase activity), GSH-Px, AR (aldose reductase) activities and MDA	6 weeks	HbA1c significantly reduced, while SOD, CAT and GPx significantly increased in DO and DI groups. Better results were found in DIO group regarding HbA1c and antioxidant enzyme activities (MDA and AR).
Ozler *et al.*, 2016 [65] Turkey	Wound healing	Experimental animals (number not informed) are divided into 3 groups: hyperbaric oxygen (HBO), ozone and control groups	Intraperitoneal injection of ozone, once a day, for 6 days at the dose of 1 mg/kg	VEGF, TGFβ1 and MMP-1 (matrix metalloproteinase-1)	1 week	VEGF of HBO group were higher than the ozone group. In HBO group, TGFβ1 were significantly higher than the other groups.
Saleh *et al.*, 2014 [69] Egypt	DM	32 rats divided into four groups (n=8 each): 1: nontreated DM; 2: DM + ozone; 3: DM + insulin; 4: DM + ozone + insulin	Intraperitoneal injection-2 ml of ozone-100 μg/ml, 2 ml/day, three applications weekly. Group 4 also received insulin	FSG (fasting serum glucose), HbA1c, TAC (total antioxidant capacity), and MAD	4 weeks	FSG and HbA1c of group 2 significantly lower than group 1. MAD of group 4 was significantly lower, and TAC was significantly higher than groups 1, 2 and 3.
Wang *et al.*, 2018 [71] United States of America	Wound healing	Diet-induced obese DM mouse model (number not informed)	Topical application of ozone oil	Cell migration, IGF1R (Insulin growth factor 1-like receptor), EGFR (epidermal growth factor receptor)	8 days	Topical ozone enhanced cell migration and activation of both EGFR and IGF1R.
Xie *et al.*, 2016 [73] China	Retinopathy	70 rats: Group A (n=10) normal control group. After inducing DM, divided into three groups: group B (no intervention), groups C (oxygen treatment) and D (ozone treatment)	Ozone clyster-50 μg/kg, twice a week	VEGF, HIF-1α and PEDF (pigment epithelium-derived factor) expression	1 month	VEGF in group D were less than group B and C. HIF-1α expression mRNA-The lowest expression in group D. PEDF mRNA expression-no significant differences were found in intra-groups.
Xie *et al.*, 2016 [72] China	Retinopathy	33 rats, 10-normal control group (blank group). After inducing DM, 12 rats-ozone group and 11 no intervention (model group)	Rectal insufflation-50 mg/mL-3 times a week	VEGF, TNF-α (Tumor necrosis factor-alpha), IL-1β (Interleukin-1 beta), IL-6 (Interleukin-6), and ICAM-1 (intercellular cell adhesion molecule-1)	1 month	Compared with the model group, each cytokine level was decreased in the ozone group. IL-6 levels fell nearly 30%, and VEGF, IL-1b, and IL-6 were close to those of the blank control group.
Yuniati *et al.* 2020 [74] Indonesia	Ulcer	60 mice divided into 6 groups (n=10 each): C-(negative control); C+ (positive control); P1 (conventional treatment); P2 (saline wound wash and 90-minute ozonated virgin coconut oil [VCO]); P3 (saline wound wash and 7-hours ozonated VCO); P4 (saline wound wash and 14-hours ozonated VCO)	Saline wound wash + Ozonated VCO applied once a day for 14 consecutive days. The wound was left open and not covered with any kind of covering	HSP90A (Heat shock protein 90A), VEGF-A (Vascular endothelial growth factor-A), EGF (Epidermal growth factor), bFGF (Basic fibroblast growth factor) and CD34 levels	14 days	The lowest HSP90Α were in the C+ group and the highest were in the P4 Group. The lowest EGF, bFGF, CD4 and VEGF-A were in the C+ group, and highest EGF were obtained in the P4.

**Abbreviations:** AOPP: Advanced oxidation protein products, DM: Diabetes mellitus, DN: Diabetic neuropathy, DNP: Diabetic nephropathy, GSH: Reduced glutathione, GSH-Px: Glutathione peroxidase, HbA1c: Glycated hemoglobin, HIF-1α: Hypoxia-inducible factor-1α, MDA: Malondialdehyde, NO2/NO3 (nitrites/nitrates), O3: Ozone, OS: Oxidative stress, OSI: Oxidative stress index, RI: Rectal insufflations, SOD: Superoxide dismutase, TGF: Transforming growth factor, VEGF: Vascular endothelial growth factor.

**Table 3:** Summary of biomarkers affected by OT and their association with the periodontal context in DM.

**Biomarkers/ Ozone Therapy Effect**	**Relevance in the Periodontal Context in DM**
**Redox balance markers:** Malondialdehyde (MDA) 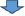 Total antioxidant capacity (TAC) 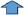 Superoxide dismutase (SOD) 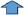 Reduced glutathione (GSH) 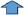 Catalase activity (CAT) 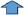 Total antioxidant status (TAS) 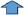 Total oxidant status (TOS) 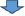	Severe tissue destruction in periodontitis is associated with excessive ROS generation, which is positively correlated in type 2 DM individuals. MDA levels increased significantly, while TAC and SOD levels decreased among periodontitis and DM individuals as compared to controls was highly significant (*p*<0.01). There was a positive correlation between the probing pocket depth and MDA levels among individuals with periodontitis and DM (r=0.566, *p*=0.003) [82]. DM individuals had higher MDA levels and decreased GSH levels in their periodontal tissues, indicating increased lipid peroxidation and impaired antioxidant defense in T2DM. These findings align with the histological changes observed, supporting the link between oxidative stress, periodontal disease, and type 2 DM [83]. There is an association between T2DM and OS-related biomarkers, particularly CAT, in individuals with periodontitis, indicating that oxidative stress plays a significant role in the development and progression of periodontitis linked to DM [84]. The instability in oxidative status (TAS and TOS) associated with DM may be a significant pathogenic factor in DM-related periodontal inflammation [85].
**Inflammatory markers:** Interleukin-1β (IL-1β) 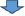 TNF- α 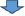 Interleukin-6 (IL-6) 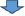 Interleukin-8 (IL-8) 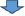 Interleukin-10 (IL-10) 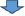 C-reactive protein (CRP) 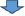	Both T2DM and periodontitis are characterized by pro-inflammatory immune activity, involving the activation of Interleukin 17 (IL-17), Interleukin 1 (IL-1), TNF-α, and Nuclear Factor Kappa Beta (NF-κB) [86]. The elevated pro-inflammatory factors (IL1-β, TNF-α, IL-6) in the gingiva of individuals with poorly controlled DM suggest a biological pathway that may aggravate periodontitis [87]. Increased circulating levels of IL-8 are associated with both types of DM in the presence of periodontal disease, suggesting IL-8's important role in the pathogenesis of T1DM, T2DM, and periodontitis. Conversely, the lowest levels of IL-10 in periodontitis indicate that periodontal inflammation itself influences serum IL-10 levels and poor glycemic control [88]. The high IL-10 expression and low IL-4 expression in the T2D group suggest an association between these markers and the impaired immune response in T2D individuals with periodontitis. Additionally, CRP inhibits osteogenesis and promotes osteoclastogenesis under diabetic and pro-inflammatory conditions, thereby disrupting alveolar bone homeostasis in periodontitis [89].
**Healing markers:** Basic fibroblast growth factor (bFGF) 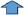 Bone morphogenic protein-2 (BMP-2) 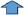 CD34 levels 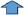 Cell migration 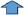 Vascular endothelial growth factor (VEGF) 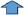 Platelet-derived growth factor (PDGF) 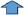	Periodontal ligament cells exposed to high glucose, which inhibits their growth, showed reduced expression of bFGF. These findings suggest that bFGF may contribute to the impaired wound healing observed in periodontal surgery of uncontrolled DM individuals [90]. DM prolongs inflammation and osteoclastogenesis in periodontitis. Through TNF, it suppresses the normal reparative process by negatively modulating factors that regulate bone. In DM animals, inhibition of TNF has been shown to reduce apoptosis, enhance proliferation of bone lining cells, and increase mRNA levels of FGF-2, TGFβ-1, BMP-2, and BMP-6 [85]. There is a significant difference in the number of CD34-positive progenitor cells between the periodontitis group and DM + periodontitis group [91]. High glucose impairs human periodontal ligament cell migration potentially through a mechanism involving the reduction of microRNA-221 and microRNA-222 expression. These effects may contribute to the impairment of periodontal healing [92]. Compared with non-DM individuals, a higher VEGF expression in gingival tissue and gingival crevicular fluid (GCF) samples was reported in DM individuals with periodontitis [93]. Levels of the growth factor PDGF, which normally increases in periodontitis, decreased in poorly controlled DM with periodontitis. These studies suggest a possible dysregulation of the normal cytokine/growth factor signaling axis in poorly controlled type 2 DM that may contribute to periodontal breakdown/diminished repair [94].
**Anti-glycemic marker:** Glycated hemoglobin (HbA1c) 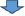	A proportional relationship was observed between PISA (periodontal inflamed surface area), clinical attachment loss, and HbA1c level in individuals with type 2 DM. A unit increase in HbA1c is associated with an increase in PISA of 130.47 mm^2^ and an increase in clinical AL of 0.182 mm [95]. Effective glycemic control improves bleeding on probing (BOP) in T2DM individuals with periodontitis by ameliorating inflammation at the gingival sites of periodontal tissue [96].

## LIST OF ABBREVIATIONS

MDA: Malondialdehyde
SOD: Superoxide Dismutase
GSH: Reduced Glutathione
CAT: Catalase
TAS: Total Antioxidant Status
TOS: Total Oxidant Status

## STANDARDS OF REPORTING

PRISMA guidelines were followed.

## SUPPLEMENTARY MATERIAL

PRISMA checklist is available as supplementary material on the publisher’s website along with the published article.

## DECLARATION

The English language of the article was improved with ChatGPT. The authors generated this text in part with GPT-4, OpenAI’s large-scale language-generation model. Upon generating draft language, the authors reviewed, edited, and revised the language to their liking and took ultimate responsibility for the content of this publication.

During the preparation of this work the authors used BioRender (https://www.biorender.com) in order to create the images.
