# Supplementary material for: The Potential of Ozone Therapy as an Adjunct to Periodontal Treatment in Diabetes Mellitus: A Biomarkers Scoping Review
Source: Curr Diabetes Rev. 2025 May 8;22(3):E15733998353396. doi: 10.2174/0115733998353396250416114716 (PMC13725158; doi:10.2174/0115733998353396250416114716)
Supplement: Supplementary file 1 [file CDR-22-3-E15733998353396_SD1.zip › CDR-22-3-E15733998353396_SD1/CDR-22-3-03-Supply.pdf]

Supplementary Material

The Potential of Ozone Therapy as an Adjunct to Periodontal Treatment in Diabetes Mellitus: A Biomarkers Scoping Review

Elisa Grillo Araújo[1,*] , Domitilla Marchiori Sant'Anna Leal de Oliveira[1] , Sérgio Bruzadelli Macedo[2] , Cristine Miron Stefani[2] and Loise Pedrosa Salles[2]

[1]*Post-Graduation in Dentistry, Faculty of Health Sciences, University of Brasilia, Brasilia, Brazil;* [2]*Department of Dentistry, Faculty of Health Sciences, University of Brasilia, Brasilia, Brazil*

Supplementary file 1 – Complete search strategy for each database.

Database	Search 2024-03-19	References
PubMed	("diabetes mellitus"[All Fields] OR "diabetes"[All Fields] OR "diabetic"[All Fields] OR "metabolic diseases"[All Fields] OR "hyperglycemia"[All Fields] OR "glucose metabolism disorders"[All Fields] OR "metabolic control"[All Fields] OR "glycated Hemoglobin A"[All Fields] OR "HbA1"[All Fields] OR "glycosylated hemoglobin A"[All Fields] OR "blood glucose"[All Fields] OR "glycemic index"[All Fields] OR "fasting blood glucose"[All Fields] OR "post-prandial blood glucose"[All Fields] OR "advanced glycation end products"[All Fields] OR "HbA1c"[All Fields] OR "glycohemoglobin A"[All Fields] OR "hemoglobin a glycosylated"[All Fields] OR (("glycated hemoglobin"[MeSH Terms] OR ("glycated"[All Fields] AND "hemoglobin"[All Fields]) OR "glycated hemoglobin"[All Fields] OR "hemoglobin a glycosylated"[All Fields]) AND ("analysis"[MeSH Subheading] OR "analysis"[All Fields])) OR "hemoglobins glycated"[All Fields] OR "glycated hemoglobin"[All Fields] OR "blood glucose metabolism"[All Fields] OR "blood glucose analysis"[All Fields] OR "diabetic patients"[All Fields] OR "glycemic control"[All Fields] OR "fasting blood glucose levels"[All Fields]) AND ("ozone"[All Fields] OR "ozone therapy"[All Fields] OR "ozontherapy"[All Fields] OR ("ozoneted"[All Fields] AND ("water"[MeSH Terms] OR "water"[All Fields] OR "watering"[All Fields] OR "water s"[All Fields] OR "watered"[All Fields] OR "waterer"[All Fields] OR "waterers"[All Fields] OR "waterings"[All Fields] OR "waters"[All Fields])) OR "ozone water"[All Fields] OR "ozone gas"[All Fields] OR ("ozoned"[All Fields] AND "oil"[All Fields]) OR "O3"[All Fields] OR "ozonated olive oil"[All Fields] OR "gaseous ozone"[All Fields] OR "ozonotherapy"[All Fields] OR (("ozonated"[All Fields] OR "ozonating"[All Fields] OR "ozonation"[All Fields] OR "ozonations"[All Fields] OR "ozone"[MeSH Terms] OR "ozone"[All Fields] OR "ozone s"[All Fields] OR "ozonization"[All Fields] OR "ozonized"[All Fields] OR "ozonizer"[All Fields]) AND ("water"[MeSH Terms] OR "water"[All Fields] OR "watering"[All Fields] OR "water s"[All Fields] OR "watered"[All Fields] OR "waterer"[All Fields] OR "waterers"[All Fields] OR "waterings"[All Fields] OR "waters"[All Fields])) OR "NBW3"[All Fields] OR "ozone ultrafine bubble water"[All Fields] OR "ozone Gel"[All Fields] OR "ozonised"[All Fields] OR "ozonized"[All Fields]) AND ("Immunology"[All Fields] OR "immunomodulation"[All Fields] OR "Interleukins"[All Fields] OR "Matrix Metalloproteinase 8"[All Fields] OR "HSP70 Heat-Shock Proteins"[All Fields] OR "NF-E2-Related Factor 2"[All Fields] OR "NF-kappa B"[All Fields] OR "HIF1A protein"[All Fields] OR "RANK Ligand"[All Fields] OR "Oxidative Stress"[All Fields] OR "Reactive Oxygen Species"[All Fields] OR "Tumor Necrosis Factor-alpha"[All Fields] OR "Interleukin-6"[All Fields] OR "Interleukin-2"[All Fields] OR "Interleukin-4"[All Fields] OR "Interleukin-10"[All Fields] OR "Interferon-gamma"[All Fields] OR "Interleukin-17"[All Fields] OR "cytokines"[All Fields])	195
Cochrane	("diabetes mellitus" OR "diabetes" OR "diabetic" OR "metabolic diseases" OR "hyperglycemia" OR "glucose metabolism disorders" OR "metabolic control" OR "glycated Hemoglobin A" OR "HbA1" OR "glycosylated hemoglobin A" OR "blood glucose" OR "glycemic index" OR "fasting blood glucose" OR "post-prandial blood glucose" OR "advanced glycation end products" OR "HbA1c" OR "glycohemoglobin A" OR "hemoglobin A, glycosylated" OR "hemoglobin A, glycosylated analysis"	9

	OR "hemoglobins glycated" OR "glycated hemoglobin" OR "blood glucose metabolism" OR "blood glucose analysis" OR “diabetic patients” OR “glycemic control” OR “fasting blood glucose levels”) AND ("ozone" OR "ozone therapy" OR "ozontherapy" OR "ozoneted water" OR "ozone water" OR "ozone gas" OR "ozoned oil" OR "O3" OR "ozonated olive oil" OR "gaseous ozone" OR "ozonotherapy" OR "ozone nano-buble water" OR "NBW3" OR "ozone ultrafine bubble water" OR "ozone Gel" OR "ozonised" OR "ozonized") AND ("Immunology" OR "immunomodulation" OR "Interleukins" OR "Matrix Metalloproteinase 8" OR "HSP70 Heat-Shock Proteins" OR "NF-E2-Related Factor 2" OR "NF-kappa B" OR "HIF1A protein" OR "RANK Ligand" OR "Oxidative Stress" OR "Reactive Oxygen Species" OR "Tumor Necrosis Factor-alpha" OR "Interleukin-6" OR "Interleukin-2" OR "Interleukin-4" OR "Interleukin-10" OR "Interferon-gamma" OR "Interleukin-17" OR "cytokines")	
Embase	('diabetes mellitus'/exp OR 'diabetes mellitus' OR 'diabetes'/exp OR 'diabetes' OR 'diabetic'/exp OR 'diabetic' OR 'metabolic diseases'/exp OR 'metabolic diseases' OR 'hyperglycemia'/exp OR 'hyperglycemia' OR 'glucose metabolism disorders'/exp OR 'glucose metabolism disorders' OR 'metabolic control'/exp OR 'metabolic control' OR 'glycated hemoglobin a'/exp OR 'glycated hemoglobin a' OR 'hba1' OR 'glycosylated hemoglobin a' OR 'blood glucose'/exp OR 'blood glucose' OR 'glycemic index'/exp OR 'glycemic index' OR 'fasting blood glucose'/exp OR 'fasting blood glucose' OR 'post-prandial blood glucose' OR 'advanced glycation end products' OR 'hba1c'/exp OR 'hba1c' OR 'glycohemoglobin a' OR 'hemoglobin a, glycosylated'/exp OR 'hemoglobin a, glycosylated' OR 'hemoglobin a, glycosylated analysis' OR 'hemoglobins glycated' OR 'glycated hemoglobin'/exp OR 'glycated hemoglobin' OR 'blood glucose metabolism' OR 'blood glucose analysis' OR 'diabetic patients'/exp OR 'diabetic patients' OR 'glycemic control'/exp OR 'glycemic control' OR 'fasting blood glucose levels') AND ('ozone'/exp OR 'ozone' OR 'ozone therapy'/exp OR 'ozone therapy' OR 'ozontherapy' OR 'ozoneted water' OR 'ozone water' OR 'ozone gas' OR 'ozoned oil' OR 'o3'/exp OR 'o3' OR 'ozonated olive oil' OR 'gaseous ozone' OR 'ozonotherapy' OR 'ozone nano-buble water' OR 'nbw3' OR 'ozone ultrafine bubble water' OR 'ozone gel' OR 'ozonised' OR 'ozonized') AND ('immunology'/exp OR 'immunology' OR 'immunomodulation'/exp OR 'immunomodulation' OR 'interleukins'/exp OR 'interleukins' OR 'matrix metalloproteinase 8'/exp OR 'matrix metalloproteinase 8' OR 'hsp70 heat-shock proteins'/exp OR 'hsp70 heat-shock proteins' OR 'nf-e2-related factor 2'/exp OR 'nf-e2-related factor 2' OR 'nf-kappa b'/exp OR 'nf-kappa b' OR 'hif1a protein'/exp OR 'hif1a protein' OR 'rank ligand'/exp OR 'rank ligand' OR 'oxidative stress'/exp OR 'oxidative stress' OR 'reactive oxygen species'/exp OR 'reactive oxygen species' OR 'tumor necrosis factor-alpha'/exp OR 'tumor necrosis factor-alpha' OR 'interleukin-6'/exp OR 'interleukin-6' OR 'interleukin-2'/exp OR 'interleukin-2' OR 'interleukin-4'/exp OR 'interleukin-4' OR 'interleukin-10'/exp OR 'interleukin-10' OR 'interferon-gamma'/exp OR 'interferon-gamma' OR 'interleukin-17'/exp OR 'interleukin-17' OR 'cytokines'/exp OR 'cytokines')	559
LIVIVO	("diabetes mellitus" OR "diabetes" OR “diabetic” OR "metabolic diseases" OR "hyperglycemia" OR "glucose metabolism disorders" OR "metabolic control" OR "glycated Hemoglobin A" OR "HbA1" OR "glycosylated hemoglobin A" OR "blood glucose" OR "glycemic index" OR "fasting blood glucose" OR "post-prandial blood glucose" OR "advanced glycation end products" OR "HbA1c" OR "glycohemoglobin A" OR "hemoglobin A, glycosylated" OR "hemoglobin A, glycosylated analysis" OR "hemoglobins glycated" OR "glycated hemoglobin" OR "blood glucose metabolism" OR "blood glucose analysis" OR “diabetic patients” OR “glycemic control” OR “fasting blood glucose levels”) AND ("ozone" OR "ozone therapy" OR "ozontherapy" OR "ozoneted water" OR "ozone water" OR "ozone gas" OR "ozoned oil" OR "O3" OR "ozonated olive oil" OR "gaseous ozone" OR "ozonotherapy" OR "ozone nano-buble water" OR "NBW3" OR "ozone ultrafine bubble water" OR "ozone Gel" OR "ozonised" OR "ozonized") AND ("Immunology" OR "immunomodulation" OR "Interleukins" OR "Matrix Metalloproteinase 8" OR "HSP70 Heat-Shock Proteins" OR "NF-E2-Related Factor 2" OR "NF-kappa B" OR "HIF1A protein" OR "RANK Ligand" OR "Oxidative Stress" OR "Reactive Oxygen Species" OR "Tumor Necrosis Factor-alpha" OR "Interleukin-6" OR "Interleukin-2" OR "Interleukin-4" OR "Interleukin-10" OR "Interferon-gamma" OR "Interleukin-17" OR "cytokines")	143

Scopus	TITLE-ABS-KEY (("diabetes mellitus" OR "diabetes" OR "diabetic" OR "metabolic diseases" OR "hyperglycemia" OR "glucose metabolism disorders" OR "metabolic control" OR "glycated Hemoglobin A" OR "HbA1" OR "glycosylated hemoglobin A" OR "blood glucose" OR "glycemic index" OR "fasting blood glucose" OR "post-prandial blood glucose" OR "advanced glycation end products" OR "HbA1c" OR "glycohemoglobin A" OR "hemoglobin A, glycosylated" OR "hemoglobin A, glycosylated analysis" OR "hemoglobins glycated" OR "glycated hemoglobin" OR "blood glucose metabolism" OR "blood glucose analysis" OR "diabetic patients" OR "glycemic control" OR "fasting blood glucose levels" AND "ozone" OR "ozone therapy" OR "ozontherapy" OR "ozoneted water" OR "ozone water" OR "ozone gas" OR "ozoned oil" OR "O3" OR "ozonated olive oil" OR "gaseous ozone" OR "ozonotherapy" OR "ozone nano-buble water" OR "NBW3" OR "ozone ultrafine bubble water" OR "ozone Gel" **OR "ozonised" OR "ozonized"** AND "Immunology" OR "immunomodulation" OR "Interleukins" OR "Matrix Metalloproteinase 8" OR "HSP70 Heat-Shock Proteins" OR "NF-E2-Related Factor 2" OR "NF-kappa B" OR "HIF1A protein" OR "RANK Ligand" OR "Oxidative Stress" OR "Reactive Oxygen Species" OR "Tumor Necrosis Factor-alpha" OR "Interleukin-6" OR "Interleukin-2" OR "Interleukin-4" OR "Interleukin-10" OR "Interferon-gamma" OR "Interleukin-17" OR "cytokines"))	219
Web of Science	TS=(("diabetes mellitus" OR "diabetes" OR “diabetic” OR "metabolic diseases" OR "hyperglycemia" OR "glucose metabolism disorders" OR "metabolic control" OR "glycated Hemoglobin A" OR "HbA1" OR "glycosylated hemoglobin A" OR "blood glucose" OR "glycemic index" OR "fasting blood glucose" OR "post-prandial blood glucose" OR "advanced glycation end products" OR "HbA1c" OR "glycohemoglobin A" OR "hemoglobin A, glycosylated" OR "hemoglobin A, glycosylated analysis" OR "hemoglobins glycated" OR "glycated hemoglobin" OR "blood glucose metabolism" OR "blood glucose analysis" OR “diabetic patients” OR “glycemic control” OR “fasting blood glucose levels”) AND ("ozone" OR "ozone therapy" OR "ozonetherapy" OR "ozoneted water" OR "ozone water" OR "ozone gas" OR "ozoned oil" OR "O3" OR "ozonated olive oil" OR "gaseous ozone" OR "ozonetherapy" OR "ozone nano-buble water" OR "nbo3" OR "ozone ultrafine bubble water" OR "ozone Gel" OR "ozonised" OR "ozonized") AND ("Immunology" OR "immunomodulation" OR "Interleukins" OR "Matrix Metalloproteinase 8" OR "HSP70 Heat-Shock Proteins" OR "NF-E2-Related Factor 2" OR "NF-kappa B" OR "HIF1A protein" OR "RANK Ligand" OR "Oxidative Stress" OR "Reactive Oxygen Species" OR "Tumor Necrosis Factor-alpha" OR "Interleukin-6" OR "Interleukin-2" OR "Interleukin-4" OR "Interleukin-10" OR "Interferon-gamma" OR "Interleukin-17" OR "cytokines"))	119
LILACS (Portuguese and Spanish)	("diabetes mellitus" OR "diabetes" OR "diabetic" OR "metabolic diseases" OR "hyperglycemia" OR "glucose metabolism disorders" OR "metabolic control" OR "glycated Hemoglobin A" OR "HbA1" OR "glycosylated hemoglobin A" OR "blood glucose" OR "glycemic index" OR "fasting blood glucose" OR "post-prandial blood glucose" OR "advanced glycation end products" OR "HbA1c" OR "glycohemoglobin A" OR "hemoglobin A, glycosylated" OR "hemoglobin A, glycosylated analysis" OR "hemoglobins glycated" OR "glycated hemoglobin" OR "blood glucose metabolism" OR "blood glucose analysis" OR "diabetic patients" OR "glycemic control" OR "fasting blood glucose levels" OR "doenças metabólicas" OR "enfermedades metabólicas" OR "Hiperglicemia" OR "hipergluce-mia" OR "Tanstornos do metabolismo de glucose" OR "Transtornos del metabolismo de la glucosa" OR "Hemoglobina A Glicada" OR "Hemoglobina A Glucada" OR "glicemia" OR "glucemia" OR "índice glicêmico" OR "índice glucémico" OR "produtos finais de glicação avançada" OR "productos finales de glicación avanzada") AND ("ozone" OR "ozone therapy" OR "ozontherapy" OR "ozone dental therapy" OR "ozoneted water" OR "ozone water" OR "ozone gas" OR "ozoned oil" OR "O3" OR "ozonated olive oil" OR "gaseous ozone" OR "ozonotherapy" OR "ozone nano-buble water" OR "NBW3" OR "ozone ultrafine bubble water" OR "ozone Gel" OR "ozonised" OR "ozonized" OR "ozônio" OR "ozono" OR "ozonioterapia" OR "ozonoterapía") AND ("Immunology" OR "immunomodulation" OR "Interleukins" OR "Matrix Metalloproteinase 8" OR "HSP70 Heat-Shock Proteins" OR "NF-E2-Related Factor 2" OR "NF-kappa B" OR "HIF1A protein" OR "RANK Ligand" OR "Oxidative Stress" OR "Reactive Oxygen Species" OR "Tumor Necrosis Factor-alpha" OR "Interleukin-6" OR "Interleukin-2" OR "Interleukin-4" OR "Interleukin-10" OR "Interferon-gamma" OR "cytokines" OR "inmunología" OR "imunologia" OR "Interleucinas" OR "Metaloproteinase 8 da Matriz" OR "Metaloproteinasa 8 de la Matriz" OR "Proteínas de Choque Térmico HSP70" OR "Proteínas HSP70 de Choque Térmico" OR "Fator 2 Relacionado a NF-E2" OR "Factor 2 Relacionado con NF-E2" OR "Subunidade p50 de NF-kappa B" OR "Subunidad p50 de NF-kappa B" OR "Ligante	07

	RANK" OR "Ligando RANK" OR "Estresse Oxidativo" OR "Estrés Oxidativo" OR "Espécies Reativas de Oxigênio" OR "Especies Reactivas de Oxígeno" OR "Fator de Necrose Tumoral alfa" OR "Factor de Necrosis Tumoral alfa" OR "Interleucina-6" OR "Interleucina-2" OR "Interleucina-4" OR "Interleucina-10" OR "Interferon gama" OR "Interferón gamma" OR "Citocinas") AND (db:("LILACS"))	
ProQuest	noft(("diabetes mellitus" OR "diabetes" OR "diabetic" OR "metabolic diseases" OR "hyperglycemia" OR "glucose metabolism disorders" OR "metabolic control" OR "glycated Hemoglobin A" OR "HbA1" OR "glycosylated hemoglobin A" OR "blood glucose" OR "glycemic index" OR "fasting blood glucose" OR "post-prandial blood glucose" OR "advanced glycation end products" OR "HbA1c" OR "glycohemoglobin A" OR "hemoglobin A, glycosylated" OR "hemoglobin A, glycosylated analysis" OR "hemoglobins glycated" OR "glycated hemoglobin" OR "blood glucose metabolism" OR "blood glucose analysis" OR "diabetic patients" OR "glycemic control" OR "fasting blood glucose levels") AND ("ozone" OR "ozone therapy" OR "ozontherapy" OR "ozoneted water" OR "ozone water" OR "ozone gas" OR "ozoned oil" OR "O3" OR "ozonated olive oil" OR "gaseous ozone" OR "ozonotherapy" OR "ozone nano-buble water" OR "NBW3" OR "ozone ultrafine bubble water" OR "ozone Gel" OR "ozonised" OR "ozonized") AND ("Immunology" OR "immunomodulation" OR "Interleukins" OR "Matrix Metalloproteinase 8" OR "HSP70 Heat-Shock Proteins" OR "NF-E2-Related Factor 2" OR "NF-kappa B" OR "HIF1A protein" OR "RANK Ligand" OR "Oxidative Stress" OR "Reactive Oxygen Species" OR "Tumor Necrosis Factor-alpha" OR "Interleukin-6" OR "Interleukin-2" OR "Interleukin-4" OR "Interleukin-10" OR "Interferon-gamma" OR "Interleukin-17" OR "cytokines"))	40
Google Scholar	("diabetes" OR "diabetes mellitus") AND ("ozone" OR "ozone therapy" OR "ozontherapy") AND ("immunology" OR "interleukins" OR "cytokines")	First 100 results

Supplementary File 2. Excluded records with reasons for exclusion.

Study	Reason for exclusion
Obolenskiy V, Protsko V, Komelyagina E, Ernolova D, Molochnikov A. Analysis of the treatment results in patients with complications of neuropathic form of diabetic foot syndrome. Journal of Wound Care. 2017;26:311-311.	Reason for exclusion: Wrong outcome.<br><br>Obs.: Biomarkers were not evaluated.
Винник, Ю С, Салмина, А Б, Теплякова, О В, Дробушевская, А И, Пожиленкова, Е А, Моргун, А В, Шапран, М В, Коваленко, А О Комбинированная озонотерапия в лечении инфекционных заболеваний мягких тканей у больных сахарным диабетом 2-го типа Khirurgiia (Mosk). Хирургия. Журнал им. НИ Пирогова - Volume 0, Issue 2, pp. 63-69 - published 2015-01-01	Reason for exclusion: Duplicate study.<br><br>Obs: Russian reference of a study already included.
Yang J, Luo K, Chen ZJ, Zhang GH, Yao X, Feng SX, Weng ZL. Clinical efficacy and metabolomic analysis of ozone major autohemotherapy for the treatment of herpes zoster. Postepy Dermatol Alergol. 2023 Oct;40(5):693-698. doi: 10.5114/ada.2023.130522.	Reason for exclusion: Wrong population.<br><br>Obs.: Non-diabetes individuals.
